# Innexin2 Regulates Limb Regeneration in *Procambarus clarkii*: Insights from Transcriptome and DNA Methylation Analyses

**DOI:** 10.3390/ani16193008

**Published:** 2026-09-24

**Authors:** Jian Li, Yude Wang, Shaojun Liu

**Affiliations:** Engineering Research Center of Polyploid Fish Reproduction and Breeding of the State Education Ministry, Hunan Normal University, Changsha 410081, China

**Keywords:** *Procambarus clarkii*, limb regeneration, WGCNA, DNA methylation, RNAi

## Abstract

Limb regeneration is one of the most fascinating phenomena in life sciences. Although *Procambarus clarkii* exhibits remarkable regenerative capabilities, the molecular mechanisms underlying its limb regeneration remain poorly understood. To address this, we investigated the transcriptome data and DNA methylation levels during the limb regeneration process in *Procambarus clarkii*. We employed fluorescence in situ hybridization (FISH), RNA interference (RNAi), and mTOR inhibitors to validate the impact of key genes and pathways. Our findings from the weighted gene co-expression network analysis (WGCNA) suggest that Innexin2 may be associated with limb regeneration. Whole-genome bisulfite sequencing (WGBS) analysis revealed changes in methylation levels between the 0 dpa and 15 dpa stages of limb regeneration, while KEGG enrichment analysis highlighted the mTOR signaling pathway. Furthermore, both Torin1 and Rapamycin, as mTOR inhibitors, demonstrated that this pathway is required for limb regeneration. Overall, our study provides insights into the molecular regulatory mechanisms underlying limb regeneration in the *Procambarus clarkii*, offering valuable data support for future research in regeneration biology.

## Simple Summary

Many animals can regrow lost body parts, a process called regeneration. The *Procambarus clarkii*, an important food species farmed in China, can regrow its claws and legs after losing them, but we do not fully understand how this works. In this study, we investigated the molecules that control limb regrowth in *Procambarus clarkii*. We found that a gene called Inx2 becomes more active during regeneration and is essential for the new limb bud to form—when we silenced this gene, almost no *Procambarus clarkii* could regrow their limbs. We also discovered that a cell growth pathway called mTOR is necessary for this process, and that chemical “switches” on the DNA (called DNA methylation) change during regeneration. Our findings help explain how *Procambarus clarkii* regrows lost limbs, which could help farmers breed healthier, faster-growing crayfish and improve aquaculture production. In the longer term, understanding how some animals regenerate body parts may also inspire new approaches in medicine for treating injuries or lost tissues in humans.

## 1. Introduction

Limb regeneration is an extremely complex regulatory process, referring to the phenomenon where an organism regrows a lost body part to restore its original form and function after suffering a physical injury. This process has long been a fascinating and captivating field of study in biology, with significant differences in regenerative capabilities observed among various animals and tissues. Some organisms, such as salamanders, possess remarkable regenerative abilities, capable of perfectly regrowing limbs [1], tails [2], and even hearts [3]. In contrast, mammals have limited regenerative capacity, but it is a research focus in regenerative medicine.

Epigenetics means that the gene expression has changed without changing the DNA sequence. This includes mechanisms such as DNA methylation, histone modifications, and gene silencing [4], and DNA methylation has been observed in various organisms, potentially influencing gene expression and growth and development [5]. Studies have shown that about 70–80% CpG sites in many fish and mice are methylated [6]. However, in crustaceans, such as *Procambarus virginalis*, only 2–3% of CpG sites undergo methylation [7]. DNA methylation plays a crucial role in the growth and development of aquatic organisms [5]. Nevertheless, the mechanisms by which DNA methylation regulates limb regeneration remain unclear.

Crustaceans generally possess regenerative abilities, often employing autotomy as a defense mechanism to escape predators [8]. After several molting cycles, they can regrow entirely new limbs [9]. While significant research has focused on appendage regeneration in vertebrates [10], the Notch signaling pathway and the TGF-β signaling pathway play significant roles in the tail regeneration of African clawed frogs and zebrafish, respectively [11,12]. Studies in invertebrates have also gained attention in recent years [13,14,15]. This ability to autotomize serves as an adaptive strategy to distract predators and facilitate escape, but the loss of limbs can impact growth and development [16]. Some studies indicate that limb loss in crustaceans can lead to reduced feeding efficiency and immune function [17]. The regeneration process typically involves several stages, including wound healing, bud formation, and growth of the new limb [18,19]. This process requires intricate regulatory mechanisms, and recent studies have identified several genes and pathways that play critical roles in crab limb regeneration [20,21,22]. However, the molecular regulatory mechanisms underlying limb regeneration in crustaceans remain poorly understood, making further research in this area highly necessary.

The red swamp crayfish (*Procambarus clarkii*), native to the central and southern United States and northeastern Mexico, was introduced to China around 1930 [23]. It exhibits excellent regenerative capabilities, strong adaptability, and high reproductive potential, making it a key economic aquatic species in China. Despite its importance, the molecular mechanisms governing limb regeneration in *Procambarus clarkii* remain understudied, which has sparked our interest. In this study, we documented the morphological characteristics of each stage of limb regeneration and conducted a stage-specific WGCNA and methylation analysis to elucidate the molecular mechanisms underlying limb regeneration in *Procambarus clarkii*.

## 2. Materials and Methods

### 2.1. Experimental Preparation and Sample Collection

All experimental *Procambarus clarkii* used in this study were obtained from Hunan Normal University. The roots of the cheliped limbs of all *Procambarus clarkii* were cut off. Tissue samples were collected from the wound sites. Samples collected on 0 dpa (day post autotomy) served as the control group. Tissue samples from the regenerating buds at the wound sites were collected at 1 dpa, 15 dpa, 30 dpa, and 45 dpa, serving as the experimental groups. The *Procambarus clarkii* was placed on ice blocks for anesthesia and sampling. The samples were immediately flash-frozen in liquid nitrogen and stored at −80 °C.

### 2.2. Histological Observation

Tissue samples were immediately fixed in 4% paraformaldehyde solution, dehydrated in a series of different alcohol concentrations, embedded in paraffin and cut to a thickness of 5 μm, then dyed with hematoxylin and eosin (H&E), and observed and photographed under a microscope (Leica Microsystems GmbH, Wetzlar, Germany).

### 2.3. RNA Sequencing and Data Analysis

For transcriptome analysis, three biological replicates were used for each regeneration stage (0, 1, 15, 30, and 45 dpa), with each replicate derived from an individual crayfish (*n* = 3). No samples were pooled across individuals. Total RNA was isolated using the Trizol method. The integrity of the RNA was assessed using the Agilent 2100 Bioanalyzer (Agilent, Santa Clara, CA, USA). mRNA was fragmented, and first-strand cDNA was synthesized. A cDNA library was constructed, and its quality was evaluated using Qubit 2.0 and the Agilent 2100 Bioanalyzer. Sequencing was performed on the Illumina Novaseq 6000 platform (Illumina, San Diego, CA, USA). Low-quality reads were filtered out, and the remaining reads were aligned to the reference genome (assembly version: GCF_020424385.1) using Hisat2 software (v2.1.0). Differentially expressed genes (DEGs) were identified using DESeq2 with the criteria of |log2(fold change)| ≥ 1 and an adjusted *p*-value (*P*adj) < 0.05, calculated using the Benjamini–Hochberg false discovery rate (FDR) correction. Functional enrichment analysis of DEGs was performed using Gene Ontology (GO) and the Kyoto Encyclopedia of Genes and Genomes (KEGG) database. Enriched GO terms and KEGG pathways with an adjusted *p*-value (*P*adj) < 0.05 (FDR-corrected) were considered statistically significant. Weighted gene co-expression network analysis (WGCNA) was conducted using the R software package (http://www.r-project.org/ (accessed on 8 July 2025)). Low-expression genes were removed, and a correlation matrix was constructed based on gene correlation coefficients. A Topological Overlap Matrix (TOM) was generated, and a hierarchical clustering tree was built. Modules associated with biological significance were analyzed based on the correlation between module characteristic genes and phenotypes. The co-expression network of the selected modules was visualized using Cytoscape (v3.8.0).

### 2.4. Whole-Genome Bisulfite Sequencing (WGBS) Analysis

For whole-genome bisulfite sequencing, three biological replicates were used for each stage (0 dpa and 15 dpa), with each replicate derived from an individual crayfish (*n* = 3); no samples were pooled. Genomic DNA was randomly fragmented to 200–400 bp by Covaris S220 (Covaris, Woburn, MA, USA). Then bisulfite treatment, PCR amplification, and DNA library construction were carried out. The quality of the library was evaluated by Agilent 5400 (Agilent Technologies, Santa Clara, CA, USA) and quantified by qPCR. Sequencing was performed on the Illumina platform (Illumina, Inc., San Diego, CA, USA). The mean genome coverage depth ranged from 13.36 to 16.35× across samples, with a mean C-site coverage of 5.4–6.6×. FastQC (v0.11.8) was used for quality control of original sequencing data. Bismark (v0.24.0) was used to align the cleaned reads to the reference genome (assembly version: GCF_020424385.1). Methylated cytosine sites were identified using a binomial test with thresholds of sequencing depth ≥ 5 and q-value ≤ 0.01. Differentially methylated regions (DMRs) were identified using the DSS software (v2.12.0), which employs a beta-binomial distribution and accounts for variance among biological replicates. DMRs were defined as regions with a minimum length of 51 bp, containing at least 4 CpG sites, with a methylation difference ≥ 0.1 and an FDR < 0.05. DMRs were annotated to gene coding region (from TSS to TES) and promoter region (2kb upstream from TSS). GO enrichment analysis was performed using GOseq. KOBAS (v3.0) was used for KEGG pathway enrichment analysis of DMR-related genes.

### 2.5. Integrated Analysis of WGBS and RNA-Seq Data

The relationship between DNA methylation and gene expression was studied by correlation analysis. Differentially methylated genes (DMGs) and differentially expressed genes (DEGs) were identified from WGBS and RNA-seq data, respectively. Scatter plots and box plots were generated as previously described. Comprehensive analysis of methylation and transcriptome data was performed. The threshold for differential methylation was set at 0.1, and the threshold for differential expression was log2|Fold change| ≥ 1. R (ggplot2) was used for data visualization. ClusterProfiler (default parameters) was employed for GO and KEGG pathway enrichment analysis of overlapping DMGs, as previously described.

### 2.6. RNA Interference Experiments

siRNAs targeting PcInx2 (5′-CGCUACCUCUGGAAGAAUA-3′; 5′-UAUUCUUCCAGAGGUAGGCG-3′) and negative control siRNA (5′-UUCUCCGAACGUGUCACGU-3′; 5′-ACGUGACACGUUCGGAGAA-3′) were synthesized by Sangon Biotech (Shanghai, China). The siRNAs were dissolved in PBS and diluted to a concentration of 0.1 μg/μL. Immediately after autotomy, each *Procambarus clarkii* in the experimental group received a single injection of 50 μL (5 μg) of PcInx2-siRNA into the base of the amputated limb using a microsyringe (Shanghai Gaoge Industrial & Trading Co., Ltd., Shanghai, China), while *Procambarus clarkii* in the control group received an equal volume (50 μL, 5 μg) of NC-siRNA under identical conditions [4]. A total of 50 *Procambarus clarkii* were used per group (*n* = 50). At 24 h and 48 h post-injection, muscle tissue from the amputation site was collected from five randomly selected *Procambarus clarkii* per group (*n* = 5). The expression level of PcInx2 was determined by quantitative real-time PCR to evaluate the silencing efficiency of PcInx2-siRNA. Bud regeneration was observed daily, and the regenerated bud length was measured until 10 days post-injection.

### 2.7. Inhibitor Injections

Torin1 (#HY-13003, MCE, Shanghai, China) and Rapamycin (#HY-10219, MCE, Shanghai, China) were dissolved in DMSO. Stock solutions of Torin1 (1 mM) and Rapamycin (2 mM) were prepared and stored at −20 °C. Injections were repeated every two days until 10 dpa. The control group was DMSO with the same volume. The solution was replaced daily until the end of the experiment to keep the same effect. Bud regeneration was observed daily, and the regenerated bud length was measured until 10 days post-injection.

### 2.8. RNA Fluorescence In Situ Hybridization (FISH)

Tissues from the amputated site were collected at 1, 15 and 30 days after amputation and fixed in 4% paraformaldehyde. The paraffin-embedded tissues were sectioned at 4 μm. A fluorescent probe was designed for the target gene sequence and labeled with FAM/Cy3 fluorescent dye. According to the instruction of the FISH kit purchased from Wuhan Seville Biotechnology Co., Ltd. (Wuhan, China), FISH operation steps were carried out. Tissue sections were incubated with probe hybridization solution overnight in a hybridization oven. After hybridization, the sections were washed with SSC buffer. A fluorescent microscope (Nikon Corporation, Tokyo, Japan) was used to observe the fluorescent signals.

### 2.9. Quantitative RT-PCR Assay

Total RNA was reverse-transcribed into cDNA by the one-step method (Takara, RR047A, Kyoto, Japan). Primers used in this experiment were designed by Primer Premier 5.0 software and synthesized by Beijing Qingke Biotechnology Co., Ltd. (Beijing, China). The primers used are listed in Table S1, and the relative expression level was verified by the 2^−ΔΔCt^ method.

## 3. Results

### 3.1. Regeneration Cycle and Morphological Changes

We observed the whole process of limb regeneration of *Procambarus clarkii*, including wound healing, bud formation and limb growth and development. At 0 dpa, the wound was formed at the amputation site (Figure 1A,B). By 1 dpa, the wound scabbed over and darkened (Figure 1A,B). Around 7 dpa, a new bud began to emerge from the wound cross-section (Figure 1A,B). By 15 dpa, the bud became distinct and prominent (Figure 1A,B). At 30 dpa, the new bud continued to grow and develop (Figure 1A,B). By 45 dpa, the new limb structure had largely formed (Figure 1A,B), although the limb remained soft and was shed during molting. Finally, by 60 dpa, a fully hardened new limb was formed (Figure 1A,B).

### 3.2. WGCNA and Modules Associated with Different Stages of Limb Regeneration

Transcriptomic analysis was carried out on the samples from different stages of limb regeneration of *Procambarus clarkii*, including immediately after limb amputation (0 dpa), scab formation (1 dpa), bud initiation (15 dpa), growth and development (30 dpa) and limb formation and growth stages (45 dpa).

Following limb autotomy, we collected tissue samples at different regeneration stages: immediately after autotomy (0 dpa), scab formation (1 dpa), bud initiation (15 dpa), growth and development (30 dpa), and limb formation and growth stages (45 dpa). We performed transcriptome analysis on these samples. Through weighted gene co-expression network analysis (WGCNA), we identified a total of 27 modules associated with the regeneration process (Figures S1–S4). After selecting those with the highest relevance and significance, we focused on eight key modules.

In Module 1, genes were enriched in chitin metabolism and protein hydrolysis, potentially regulating growth and immune responses (Figure 2 and Figure S5A). Module 2 contained genes enriched in RNA processing, splicing, and modification, suggesting activation of protein synthesis and cellular function regulation, which may promote cell differentiation and proliferation (Figure 2 and Figure S5B). Module 3 was enriched with genes involved in the cell cycle and cell cycle processes, indicating activation during cell division and muscle development (Figure 2 and Figure S6A). Module 4 included genes enriched in protein hydrolysis and degradation metabolism, processes crucial for immune responses in crustaceans, such as the role of melanization in wound healing (Figure 2 and Figure S6B). Module 5 was also enriched with genes involved in chitin metabolism (Figure 2 and Figure S7A). Module 6 included genes involved in transmembrane transport and lipid transport, participating in signal transduction and cell communication, ensuring normal cellular physiology (Figure 2 and Figure S7B). Module 7 was enriched with genes involved in ATP metabolic processes and various phosphate metabolism processes, providing energy for cell growth and proliferation (Figure 2 and Figure S8A). Module 8 included genes involved in signal transduction and protein dephosphorylation, ensuring normal cell growth and environmental adaptation (Figure 2 and Figure S8B).

GO enrichment analysis of DEGs in limb regeneration revealed that different biological processes were involved in different stages of limb regeneration (Figure S9). The results showed that 1 dpa was upregulated in genes related to protein modification and signal transduction and genes involved in cell cycle regulation. Genes related to ATP biosynthesis and energy metabolism were upregulated in limb regeneration for 15 dpa. At 30 dpa, genes involved in the regulation of gene expression and cell communication were upregulated. Finally, at 45 dpa, genes associated with the cell cycle and developmental processes were involved, likely playing a role in regulating tissue growth and development (Figure S9).

We identified key genes within these modules, with Innexin emerging as a central hub gene. We determined the chromosome position of 17 Innexin genes (Figure S10). To investigate the expression of Innexin genes during limb regeneration in *Procambarus clarkii*, we analyzed the RNA-seq data and observed an interesting pattern. Compared to 0 dpa, Innexin genes were upregulated at 1 dpa, and this upregulation progressively increased throughout the regeneration process. By 45 dpa, eight Innexin genes showed significant upregulation (*P*adj < 0.05). Among them, Inx2 (ID: 123773347) was upregulated most significantly during the limb regeneration of *Procambarus clarkii* (*P*adj < 0.001) (Figure 3A). These findings suggest that Inx2 may play a critical and essential role in *Procambarus clarkii* limb regeneration after autotomy. Through Cytoscape (v3.8.0) analysis, we found that Inx2, as a hub gene, interacts with MEF2 and ELF5 (Figure 3D). ELF5 is a key regulatory factor in the mTOR signaling pathway, controlling the initiation of protein synthesis. MEF2 regulates the activation of the mTOR signaling pathway and promotes cell anabolism and proliferation, which is very important in the process of cell growth and development. Based on these findings, we propose that Inx2 may be functionally associated with the mTOR signaling pathway, although this link requires further investigation.

### 3.3. RNA Fluorescence In Situ Hybridization (FISH) Analysis

The expression of Inx2 in the limb regeneration of *Procambarus clarkii* was explored by RNA FISH. At 1 dpa, Inx2 was predominantly distributed in the epidermal cells. By 15 dpa and 30 dpa, Inx2 was localized to the joint regions of the buds and the base of the buds (Figure 3C). These results suggest that Inx2 may play a role in regulating bud formation and growth during *Procambarus clarkii* limb regeneration.

### 3.4. RNA Interference and mTOR Inhibitor Experiments

To explore the function of Inx2, RNA interference experiments were conducted. The results showed that 24 h and 48 h after Inx2-RNAi injection, the expression level of Inx2 was significantly reduced compared to NC-RNAi (*p* < 0.0001) (Figure 3B), indicating successful gene silencing. Survival was monitored daily throughout the experiment. In the Inx2-RNAi group, 27 of 50 animals died after 7 days post-injection, whereas seven of 50 control animals died during the same period. Ten days after Inx2-RNAi injection, we observed that 72% of the control group (36 out of 50) developed new buds, while only 6% of the experimental group (three out of 50) formed new buds. When mortality was accounted for by analyzing only surviving animals, the bud formation rate in the Inx2-RNAi group (three out of 23, 13.0%) remained significantly lower than that in the control group (36 out of 43, 83.7%; *p* < 0.0001). These results indicate that the impaired bud formation in the Inx2-RNAi group is not merely a consequence of mortality but reflects a specific defect in limb regeneration.

To investigate the importance of the mTOR pathway in *Procambarus clarkii* limb regeneration, we conducted mTOR inhibitor experiments using rapamycin and Torin1. The regenerated bud lengths in the experimental groups (injected with rapamycin and Torin1) were significantly shorter than those in the control group at 10 dpa (0.23 ± 0.06 mm and 0.16 ± 0.07 mm, respectively, compared to 0.67 ± 0.24 mm in the control group, *p* < 0.01). Our results demonstrate that the mTOR signaling pathway is required for limb regeneration and that Inx2 is essential for bud formation in *Procambarus clarkii*. However, whether Inx2 exerts its function through the mTOR pathway remains to be determined.

### 3.5. DNA Methylation Analysis

To investigate the potential role of DNA methylation during the transition from wound healing to bud formation, we performed WGBS on regenerating limb tissues at 0 dpa (immediately after autotomy) and 15 dpa (bud formation stage). The average methylation levels for the 0 dpa group were 98.99% for CG, 0.24% for CHG, and 0.74% for CHH, while for the 15 dpa group, the levels were 98.96% for CG, 0.26% for CHG, and 0.77% for CHH. Compared to CHG and CHH contexts, CG contexts exhibited the highest methylation levels (Figure 4A–C). We analyzed the methylation level distribution of three sequence contexts (CG, CHG, CHH) in CpG islands (CGI), gene functional elements, and the 2kb upstream regions of genes (Figure 4D–F). In total, 75943 CG differentially methylated regions (DMRs), 1167 CHG DMRs, and 3122 CHH DMRs were identified between the 0 dpa and 15 dpa groups (Figure 4G). We identified 1601 CG_DMR genes, 974 CHH_DMR genes, and 363 CHG_DMR genes across the three methylation contexts (Figure 4H). Subsequent DNA methylation analysis of the two muscle groups primarily focused on CG contexts. For DMR-anchored regions (such as promoters, exons, introns, CGI, CGI shores, repeats, TSS, TES, etc.), we differentiated high-methylation DMRs (hyper) from low-methylation DMRs (hypo) and presented the results in a statistical graph (Figure 4I). Low methylation was more prevalent than high methylation. GO and KEGG enrichment analyses of DMR-associated genes revealed that GO was significantly enriched in the “non-membrane-bounded organelle (Figure 4J),” while KEGG pathways were mainly enriched in the “mTOR signaling pathway,” “ribosome,” and “RNA polymerase (Figure 4K).”

### 3.6. Integrated Analysis of DNA Methylation and Gene Expression

We conducted an integrated analysis of DNA methylation and gene expression data from the 0 dpa and 15 dpa groups. A volcano plot was used to visualize the expression levels of differentially expressed genes (DEGs) and the methylation levels of differentially methylated regions (DMRs) (Figure 5A). Venn diagrams were generated to show the overlap between DEGs, DMRs, and DMPs in the two groups (Figure 5B). The relationship between methylation levels and gene expression levels was analyzed, revealing that gene body methylation was positively correlated with gene expression, while promoter methylation was negatively correlated (Figure 5C).

GO enrichment analysis of CG-type DMR-targeted DMGs revealed that genes involved in RNA transport, protein modification processes, and protein phosphorylation were activated, suggesting the activation of protein synthesis and cellular processes that promote cell differentiation and proliferation (Figure 5D). Conversely, genes involved in hydrolase activity and phosphorylation were suppressed, which is crucial for ensuring the orderly progression of regeneration.

The integrated analysis of RNA-seq and methylation data revealed significant changes in the mTOR pathway, both in terms of gene expression and methylation. The use of mTOR inhibitors, such as rapamycin and Torin1, demonstrated that the mTOR pathway is essential for *Procambarus clarkii* limb regeneration, as the number of regenerated buds in the experimental group was significantly smaller than in the control group. This indicates that the mTOR pathway is indispensable for limb regeneration in *Procambarus clarkii*.

Additionally, the RNA-seq data showed upregulation of genes such as Pi3K21B, IRS1, and FZD5 in the mTOR pathway, while methylation data revealed methylation changes in Wnt-16 and PIK3C genes in the same pathway. These findings highlight the potential association between DNA methylation and the mTOR pathway during limb regeneration.

In summary, the integrated analysis of DNA methylation and gene expression data provided comprehensive insights into the epigenetic regulation of limb regeneration in *Procambarus clarkii*. The findings underscore the potential role of DNA methylation in gene expression and confirm that the mTOR pathway is required for the regenerative process.

## 4. Discussion

Limb regeneration in *Procambarus clarkii* involves the regeneration of appendages composed of jointed segments, which are essential for basic movement [24]. Autotomy, the voluntary shedding of a limb, serves as a defense mechanism to escape predator attacks [25]. However, limb regeneration is a complex biological process. The regeneration process begins with wound healing, mainly for plasma coagulation at the wound site [26,27]. Subsequently, a blastema forms at the wound site, consisting of pluripotent cells that aggregate and undergo continuous proliferation to form a bud. These cells have the potential to differentiate into various cells needed for limb regeneration [28,29,30,31]. During the growth and differentiation phase, the bud continues to grow until a new limb is fully formed [32,33].

Innexins are gap junction proteins found in almost all invertebrates [34]. They can pass through some ions and small metabolites, and are also regulated by factors such as pH [35], calcium [36], and voltage [37]. Innexins are very important in the process of cell proliferation and differentiation, and are also crucial to the normal development of tissues [38,39]. In this study, WGCNA identified Inx2 as a core hub gene, suggesting its crucial and necessary role in the limb regeneration process of *Procambarus clarkii* after autotomy. Cytoscape analysis revealed that Inx2 interacts with MEF2 and ELF5. MEF2 regulates the activation of the mTOR signaling pathway, a mechanism that promotes anabolic metabolism and proliferation, which are critical for cellular growth and development [40]. Similarly, ELF5 is a key regulator in the mTOR signaling pathway, which controls the initiation of protein synthesis [41]. RNA interference confirmed that Inx2 is essential for bud formation in *Procambarus clarkii*: siRNA knockdown markedly reduced Inx2 expression at 24 h and 48 h post-injection, and bud formation was almost completely abolished (6%, 3/50) compared with the control group (72%, 36/50). These results indicate that Inx2 is required for the initiation or early growth of the regenerating bud. In crustaceans, limb regeneration is intrinsically linked to the molting cycle. In the present study, no apparent difference in molting frequency was observed between the surviving Inx2-siRNA and control animals during the 10-day observation period. However, given the short observation period compared with the *Procambarus clarkii* molting cycle, we cannot exclude potential effects of Inx2 knockdown on molting over longer time scales. Limb regeneration involves complex regulatory mechanisms that coordinate various cell types and molecular processes to achieve successful regeneration [42]. The mTOR pathway is involved in many physiological processes [43] and it also plays a key role in zebrafish caudal fin regeneration [44] and newt organ regeneration. However, the activation of mTOR during the early wound healing phase in *Procambarus clarkii* limb regeneration has not been previously reported. In this study, the molecular mechanism of limb regeneration of *Procambarus clarkii* was discussed. The results showed that the mTOR signaling pathway played a key role in limb regeneration. To further validate whether mTOR is essential for limb regeneration, we used two mTOR inhibitors, rapamycin and Torin1. Rapamycin, a natural product [45], and Torin1, a synthetic molecule [46], were chosen for their inhibitory effects on mTOR, with rapamycin being less potent than Torin1. We simultaneously used both inhibitors to study their effects on limb regeneration. Our observations during the regeneration process revealed that mTOR inhibition significantly impaired limb regeneration, as the base of the bud in the experimental group could not grow normally, resulting in a much lower growth rate compared to the control group. These findings suggest that the mTOR signaling pathway is required for normal limb regeneration in *Procambarus clarkii* after autotomy. However, whether the pathway is actively upregulated (activated) during regeneration, and how it is mechanistically linked to upstream regulators such as Inx2, cannot be concluded from the present data and warrants further investigation.

DNA methylation has been reported to be associated with growth and development [47,48,49,50], but research on its role in limb regeneration and development remains limited. Some studies have reported that DNA methylation is associated with the early stages of salamander limb regeneration and cell fate determination [51]. In this study, we compared genome-wide DNA methylation profiles between the amputation stage (0 dpa) and the bud formation stage (15 dpa) of limb regeneration in *Procambarus clarkii*. The choice of these two stages was based on the critical transition from wound healing to blastema formation. In this study, the whole-genome CpG methylation levels in *Procambarus clarkii* ranged from 9.9% to 11.93%. Compared to CHG and CHH contexts, CG contexts exhibited the highest methylation levels, approximately 98.9%. KEGG analysis showed that DMR-associated genes were primarily enriched in the mTOR signaling pathway, with Wnt-16 and PIK3C genes undergoing methylation. This indicates that the mTOR signaling pathway is required for limb regeneration in *Procambarus clarkii*.

In summary, based on the temporal sequence of limb regeneration, our gene co-expression network analysis identified Inx2 as a key core hub gene that undergoes significant changes during regeneration. RNAi of Inx2 resulted in death and failure to regenerate buds in most individuals, while the majority of control individuals successfully regenerated new buds. These findings demonstrate that Inx2 is essential for limb regeneration and that mTOR signaling is required for this process. Whether Inx2 functions through the mTOR pathway remains to be determined; the predicted interaction between Inx2 and the mTOR-related regulators MEF2 and ELF5 provides a hypothesis that warrants direct experimental validation in future studies. This discovery provides critical insights into the molecular mechanisms underlying *Procambarus clarkii* limb regeneration after autotomy.

## 5. Conclusions

In this study, we characterized the morphological progression of limb regeneration in *Procambarus clarkii* and integrated transcriptomic, methylomic, and functional analyses to explore the underlying molecular basis. Weighted gene co-expression network analysis identified Inx2 as a key hub gene whose expression increased progressively during regeneration, and RNA interference demonstrated that Inx2 is essential for bud formation. Pharmacological inhibition further showed that the mTOR signaling pathway is required for normal limb regeneration. In addition, whole-genome bisulfite sequencing revealed DNA methylation changes between the 0 dpa and 15 dpa stages, with DMR-associated genes enriched in the mTOR pathway, suggesting a potential association between DNA methylation and this pathway. However, whether Inx2 exerts its function through mTOR signaling remains to be determined and warrants direct experimental validation in future studies. Together, these findings provide new insights into the molecular mechanisms underlying limb regeneration in crustaceans and offer a foundation for future research in regeneration biology and aquaculture.

## Figures and Tables

**Figure 1:**
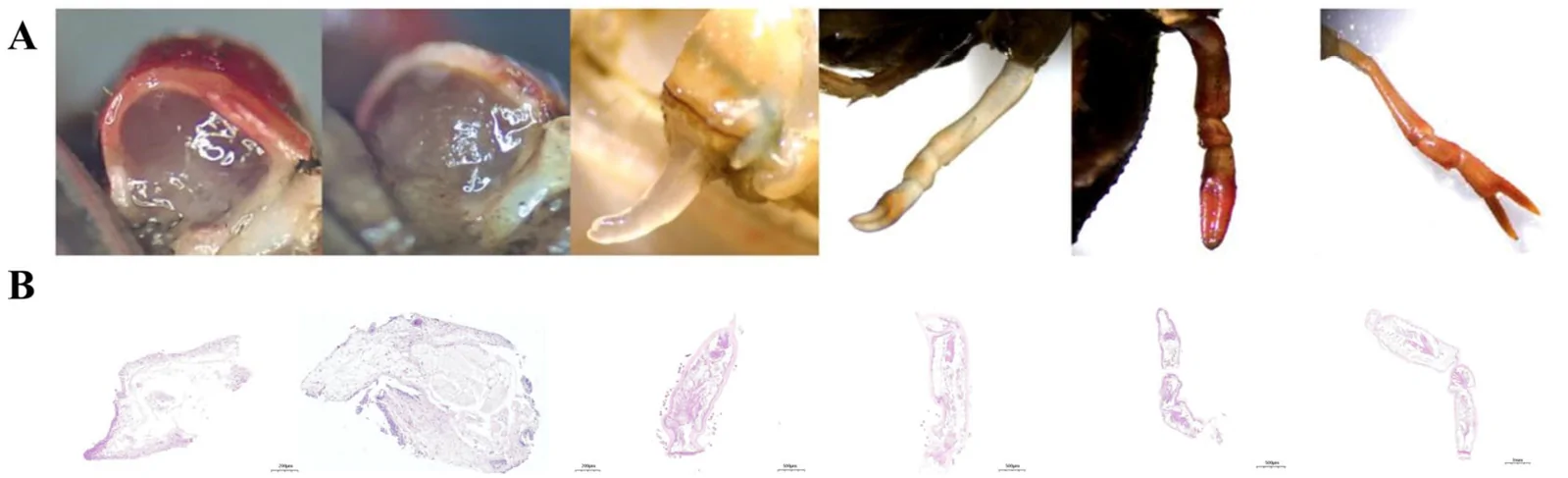
The limb regeneration process of *Procambarus clarkii*. (**A**) Observation of the entire regeneration process of the *Procambarus clarkii*. (**B**) HE staining at six time points (0, 1, 15, 30, 45, and 60 dpa).

**Figure 2:**
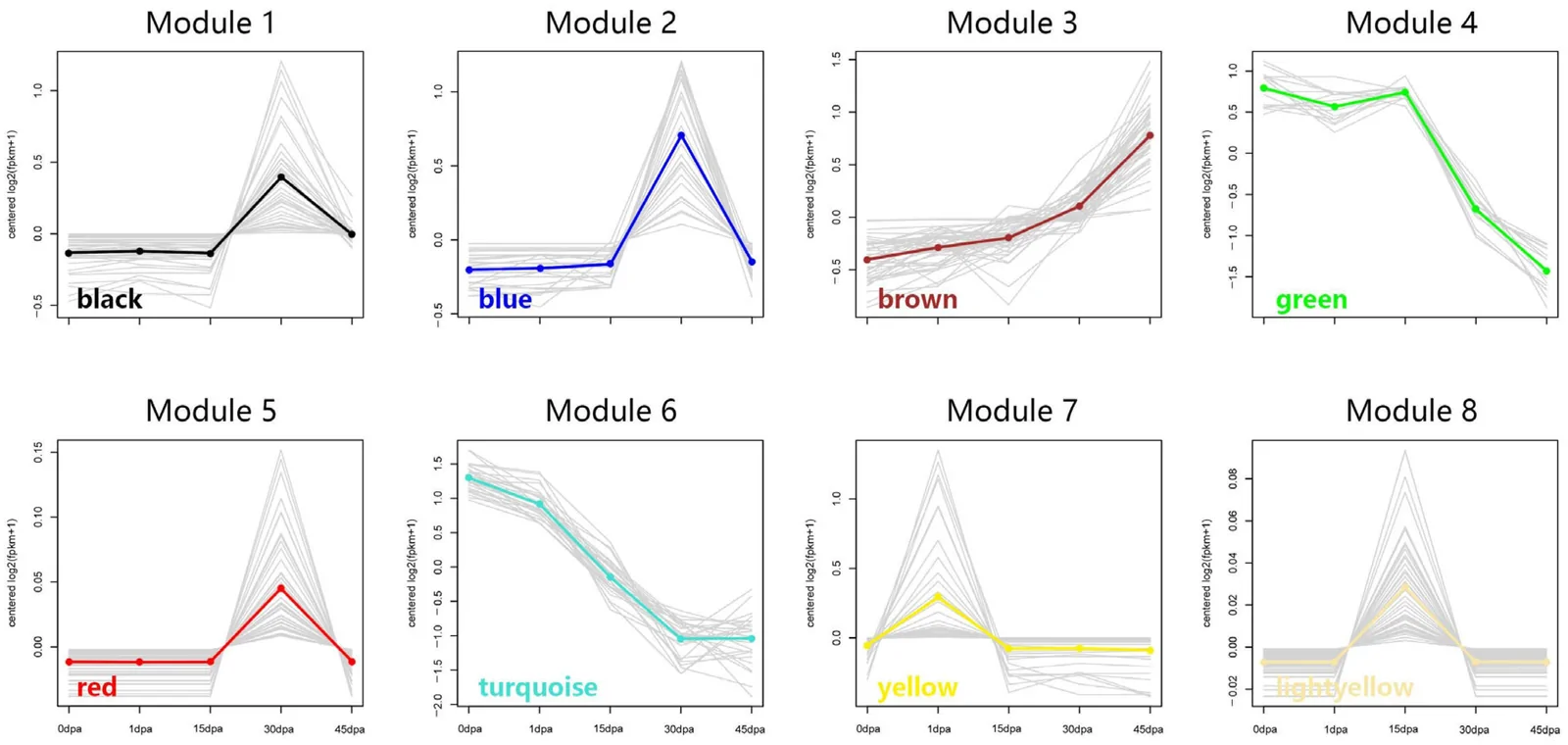
The co-expression network during the limb regeneration process of the crayfish was analyzed. Through WGCNA analysis, the expressions of eight modules (Modules 1 to 8) were correlated with different regeneration stages.

**Figure 3:**
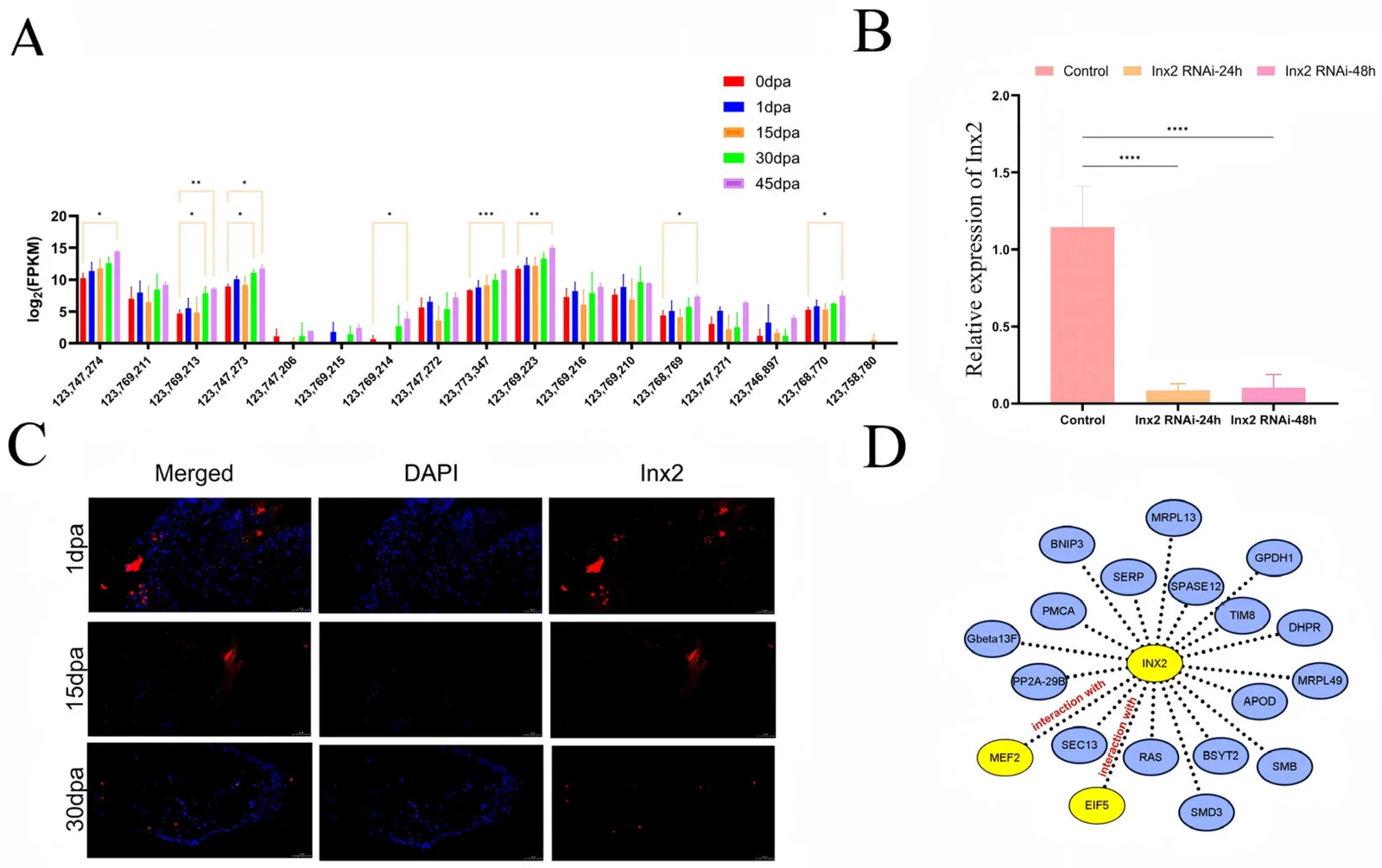
Innexin 2 regulates limb regeneration of *Procambarus clarkii*. (**A**) Sixteen Innexin genes were expressed during limb regeneration. (**B**) The expression of Inx2 gene after RNAi. (**C**) Fluorescence in situ hybridization (*FISH*) of Inx2 at 1, 15 and 30 dpa. (**D**) Co-expression network showing genes co-expressed with Inx2. * (*p* < 0.05), ** (*p* < 0.01), *** (*p* < 0.001), and **** (*p* < 0.001).

**Figure 4:**
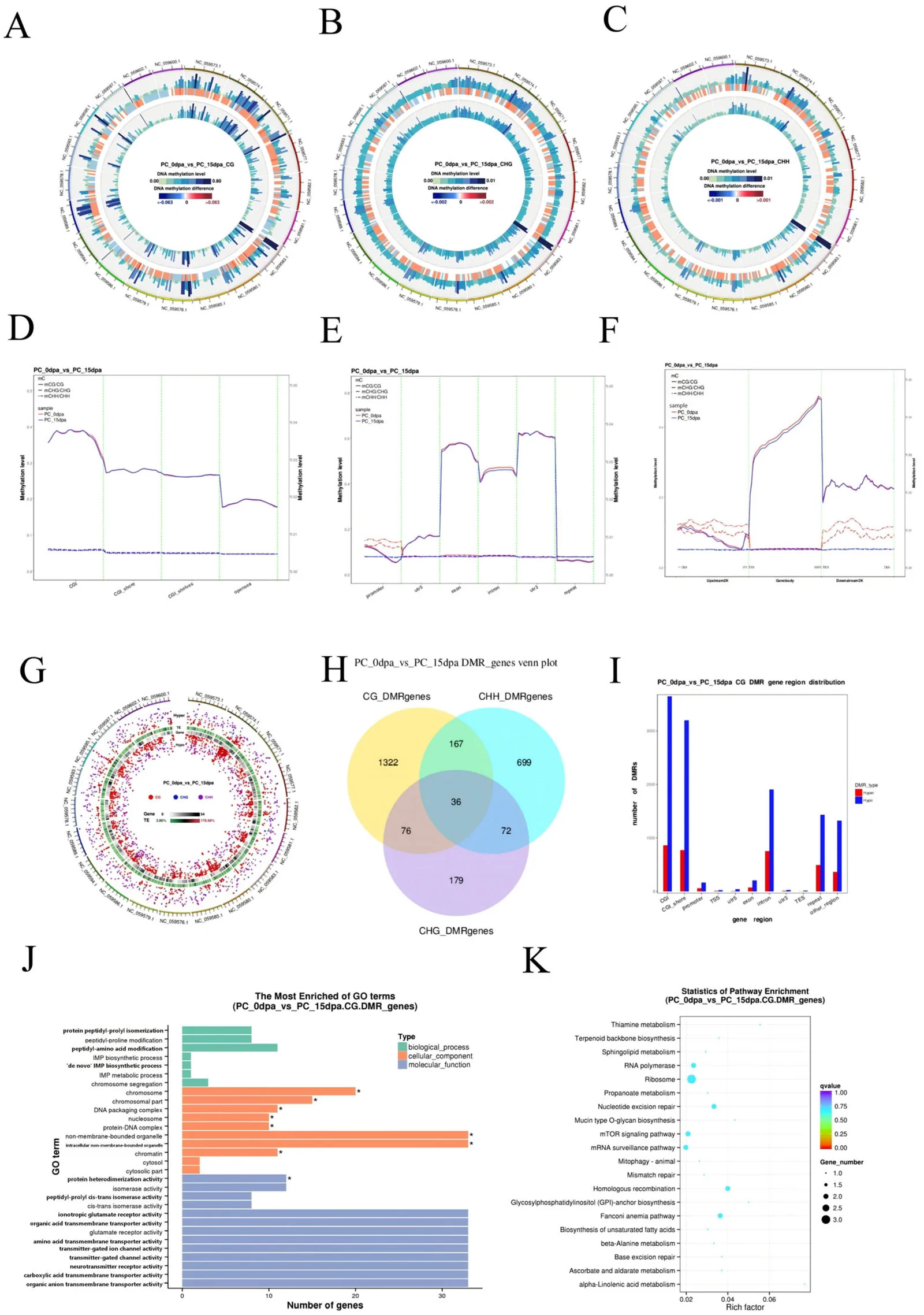
Comparative analysis of DNA methylation was conducted between 0 dpa and 15 dpa groups. (**A**–**C**) Circos plots of methylation levels for three types of sequences (CG, CHG, CHH). (**D**) Distribution of methylation levels in three sequence environments (mCG, mCHG, mCHH) on CGI region. (**E**,**F**) Density plots of methylation levels in different functional gene elements. (**G**) Circos display of three cytosines in the 0 dpa and 15 dpa groups. (**H**) Venn diagram of DMR-associated genes. (**I**) Distribution of low- and high-methylation DMRs in different functional gene elements between the 0 dpa and 15 dpa groups. (**J**,**K**) GO and KEGG enrichment analyses of DMR-targeted differentially methylated genes (DMGs). * (*p* < 0.05).

**Figure 5:**
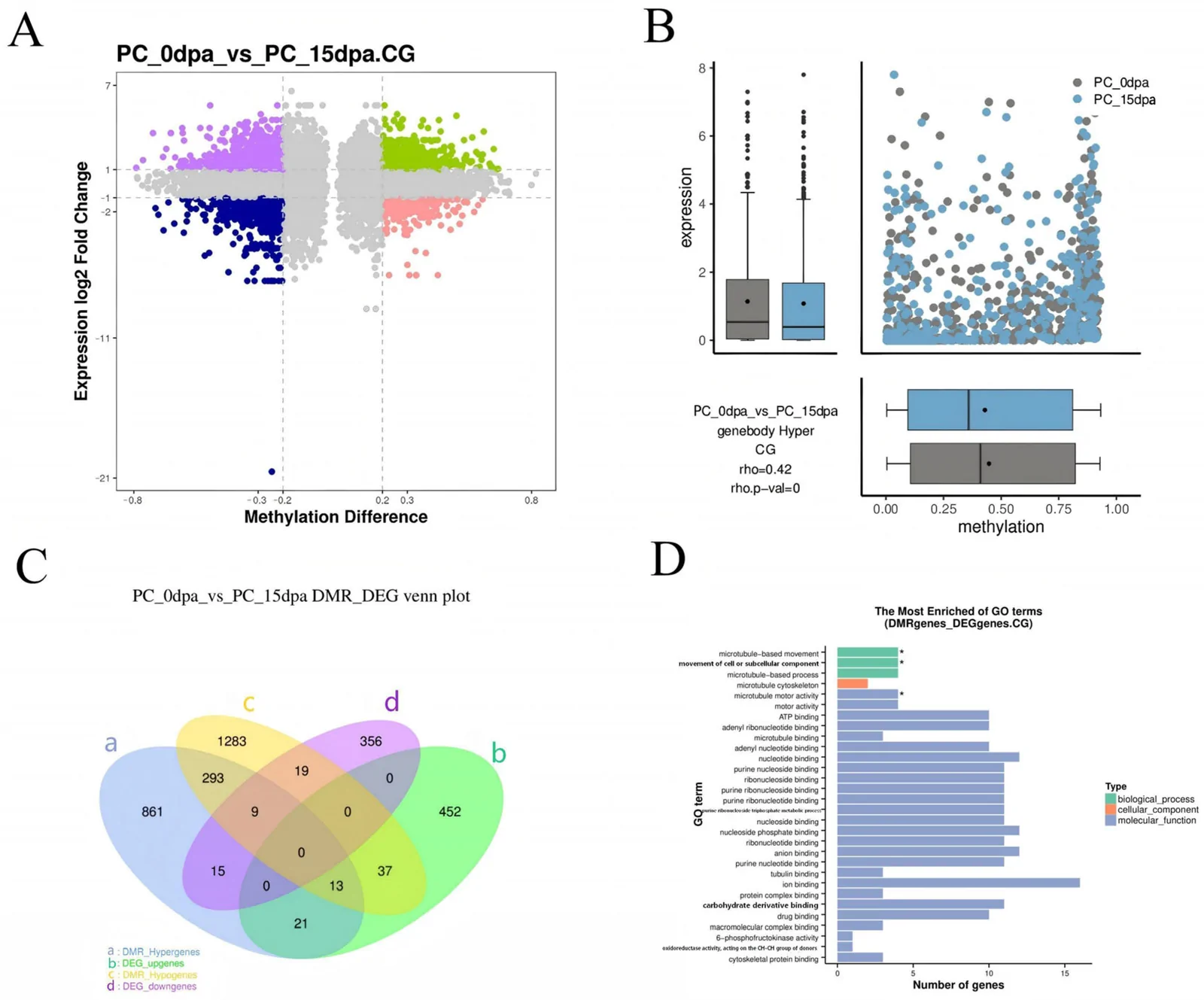
Integrative analysis of WGBS and transcriptomics in the muscles of the 0 dpa and 15 dpa groups. (**A**) Correlation between combined DMR differential methylation levels and the expression levels of the corresponding transcriptome DEGs. (**B**) Relationship between methylation level and related gene expression. (**C**) Venn diagram of DEG expression and DMP target DMGs in muscles between the 0 dpa and 15 dpa groups. (**D**) GO enrichment of the overlapping DMR-targeted DMGs with CG types. * (*p* < 0.05).

## Data Availability

The data from the study are available from the corresponding authors upon reasonable request.

## Supplementary Materials

The following supporting information can be downloaded at https://www.mdpi.com/article/10.3390/ani16193008/s1. Figure S1. Correlation between the fitting index and soft threshold (left). Figure S2. The relationship between common module characteristic genes and regeneration process was identified by WGCNA analysis. We determined the co-expression module of gene 227. With the time change of limb regeneration, the specific module of *Procambarus clarkii* showed strong correlation with different limb regeneration stages (0 dpa, 1 dpa, 15 dpa and 30 pad). Dpa: days post autotomy. Figure S3. Differentially expressed gene *Procambarus clarkii* during limb regeneration. Thermogram of differentially expressed genes during limb regeneration. Figure S4. Identification of Cluster Tree Based on Co-expression Topology Overlapping Display Module by WGCNA. A total of 27 modules were identified, and different horizontal color bars represent different modules of co-expressed genes. Figure S5. GO enrichment analysis for co-expressed genes in Module 1 (A) and Module 2 (B) from Figure 2 by WGCNA analysis. Figure S6. GO enrichment analysis for co-expressed genes in Module 3 (A) and Module 4 (B) from Figure 2 by WGCNA analysis. Figure S7. GO enrichment analysis for co-expressed genes in Module 5 (A) and Module 6 (B) from Figure 2 by WGCNA analysis. Figure S8. GO enrichment analysis for co-expressed genes in Module 7 (A) and Module 8 (B) from Figure 2 by WGCNA analysis. Figure S9. GO enrichment analysis is carried out on representative biological process categories of up-regulated genes. 1 dpa, 15 dpa, 30 dpa and 45 dpa during limb regeneration. dpa: day post autotomy. Figure S10. Chromosome distribution of 17 Innexin genes in the genome of *Procambarus clarkii*. Table S1. Primer sequences used for RT-qPCR analysis for related genes in this study.

## References

[1] Young H.E., Bailey C.F., Dalley B.K. (1983). Gross morphological analysis of limb regeneration in postmetamorphic adult *Ambystoma*. Anat. Rec..

[2] Echeverri K., Tanaka E.M. (2002). Ectoderm to Mesoderm Lineage Switching During Axolotl Tail Regeneration. Science.

[3] Godwin J.W., Debuque R., Salimova E., Rosenthal N.A. (2017). Heart regeneration in the salamander relies on macrophage-mediated control of fibroblast activation and the extracellular landscape. npj Regen. Med..

[4] Zhu L., Yuan G., Jiang X., Zhang J., Zhao X., Pei C., Li L., Kong X. (2020). Molecular characterization of Rab5 and Rab6, and their involvement in innate immunity in red swamp crayfish *Procambarus clarkii*. Aquaculture.

[5] Liu Z., Zhou T., Gao D. (2022). Genetic and epigenetic regulation of growth, reproduction, disease resistance and stress responses in aquaculture. Front. Genet..

[6] Feng S., Cokus S.J., Zhang X., Chen P.-Y., Bostick M., Goll M.G., Hetzel J., Jain J., Strauss S.H., Halpern M.E. (2010). Conservation and divergence of methylation patterning in plants and animals. Proc. Natl. Acad. Sci. USA.

[7] Vogt G. (2022). Studying phenotypic variation and DNA methylation across development, ecology and evolution in the clonal marbled crayfish: A paradigm for investigating epigenotype-phenotype relationships in macro-invertebrates. Sci. Nat..

[8] Maginnis T.L. (2006). The costs of autotomy and regeneration in animals: A review and framework for future research. Behav. Ecol..

[9] Das S. (2015). Morphological, Molecular, and Hormonal Basis of Limb Regeneration across Pancrustacea. Integr. Comp. Biol..

[10] Stocum D.L., Cameron J.A. (2011). Looking proximally and distally: 100 years of limb regeneration and beyond. Dev. Dyn. An. Off. Publ. Am. Assoc. Anat..

[11] Beck C.W., Christen B., Slack J.M.W. (2003). Molecular Pathways Needed for Regeneration of Spinal Cord and Muscle in a Vertebrate. Dev. Cell.

[12] Meshram D.D., Liu Y., Worth M., Zhang C., Carney T.J., Roehl H.H. (2025). TGF-beta coordinates changes in Keratin gene expression during complex tissue regeneration. Sci. Rep..

[13] Bando T., Ishimaru Y., Kida T., Hamada Y., Matsuoka Y., Nakamura T., Ohuchi H., Noji S., Mito T. (2013). Analysis of RNA-Seq data reveals involvement of JAK/STAT signalling during leg regeneration in the cricket *Gryllus bimaculatus*. Development.

[14] Lee A.K., Sze C.C., Kim E.R., Suzuki Y. (2013). Developmental coupling of larval and adult stages in a complex life cycle: Insights from limb regeneration in the flour beetle, *Tribolium castaneum*. EvoDevo.

[15] Li J., Zuo J., Lv X., Ma J., Li X., Fu S., Sun J. (2023). Hedgehog signaling is essential in the regulation of limb regeneration in the Chinese mitten crab, *Eriocheir sinensis*. Fish. Shellfish Immunol..

[16] Maginnis T.L., Masis I., Degerstedt S.G., Luethke T.J., Ortman M. (2015). The Effects of Cheliped Autotomy and Regeneration on Aggression in Purple Shore Crabs (Hemigrapsus nudus).

[17] Gong J., Huang C., Yu K., Li S., Zeng C., Ye H. (2022). The effects of feeding ration and cheliped autotomy on the growth and expression of ecdysteroid receptor in early juvenile mud crabs, *Scylla paramamosain*. Aquac. Rep..

[18] Farhadi A., Xue L., Xie J., Zhao Q., Khojasteh S.M.B., Kor G. (2026). Cheliped regeneration in red-claw crayfish (*Cherax quadricarinatus*): Molecular mechanisms and effects on growth, molting, and survival. Aquaculture.

[19] Wang J., Chen X., Hou X., Wang J., Yue W., Huang S., Xu G., Yan J., Lu G., Michael H. (2022). “Omics” data unveil early molecular response underlying limb regeneration in the Chinese mitten crab, *Eriocheir sinensis*. Sci. Adv..

[20] Shinji J., Gotoh H., Miyanishi H., Lavine M.D., Lavine L.C. (2019). The activin signaling transcription factor Smox is an essential regulator of appendage size during regeneration after autotomy in the crayfish. Evol. Dev..

[21] Wang Y., Huang X., Zhou Q., Tian Y., Zuo J., Yuan Z., Liu Y., Li J., Sun J. (2023). Hippo Signaling Regulates Blastema Formation During Limb Regeneration in Chinese Mitten Crab (*Eriocheir sinensis*). Mar. Biotechnol..

[22] Liu L., Fu Y., Zhu F., Mu C., Li R., Song W., Shi C., Ye Y., Wang C. (2018). Transcriptomic analysis of *Portunus trituberculatus* reveals a critical role for WNT4 and WNT signalling in limb regeneration. Gene.

[23] Huang J., Tang S., Cai F., Lin Y., Wu Z. (2017). Microsatellite evidence of dispersal mechanism of red swamp crayfish (Procambarus clarkii) in the Pearl River basin and implications for its management. Sci. Rep..

[24] Anger K., Harzsch S., Thiel M. (2020). Developmental Biology and Larval Ecology: The Natural History of the Crustacea.

[25] He J., Wu X., Cheng Y. (2016). Effects of limb autotomy on growth, feeding and regeneration in the juvenile *Eriocheir sinensis*. Aquaculture.

[26] Kifayatullah M., Golara K., Antonín K., Pavel K., Hamid N. (2022). Hemocyte coagulation and phagocytic behavior in early stages of injury in crayfish (Arthropoda: Decapoda) affect their morphology. Dev. Comp. Immunol..

[27] Farhadi A., Xue L., Zhao Q., Tan K. (2024). An overview of recent progress in the molecular mechanisms and key biological macromolecules involved in limb regeneration of decapods. Int. J. Biol. Macromol..

[28] Musgrove L., Bhojwani A., Hyde C., Glendinning S., Nocillado J., Russell F.D., Ventura T. (2024). Transcriptomic Analysis across Crayfish (*Cherax quadricarinatus*) Claw Regeneration Reveals Potential Stem Cell Sources for Cultivated Crustacean Meat. Int. J. Mol. Sci..

[29] Tetsuya B., Hitoshi Y., Harukazu N. (2018). Wound repair, remodeling, and regeneration. Dev. Growth Differ..

[30] Atsushi K. (2009). Stem cell system in tissue regeneration in fish: Stem cell system in fish regeneration. Dev. Growth Differ..

[31] Sara S., Nuno A., Anabela B.-B., Mariana F., Mariana S., Joaquín L., Henry R., Leonor C.M., António J. (2011). Differentiated skeletal cells contribute to blastema formation during zebrafish fin regeneration. Development.

[32] Zhong J., Jing A., Zheng S., Li S., Zhang X., Ren C. (2023). Physiological and molecular mechanisms of insect appendage regeneration. Cell Regen..

[33] Hopkins P.M. (1993). Regeneration of walking legs in the fiddler crab *Uca pugilator*. Am. Zool..

[34] Skerrett I.M., Williams J.B. (2017). A structural and functional comparison of gap junction channels composed of connexins and innexins. Dev. Neurobiol..

[35] Abrams C.K., Freidin M.M., Verselis V.K., Bennett M.V.L., Bargiello T.A. (2001). Functional alterations in gap junction channels formed by mutant forms of connexin 32: Evidence for loss of function as a pathogenic mechanism in the X-linked form of Charcot-Marie-Tooth disease. Brain Res..

[36] Bennett B.C., Purdy M.D., Baker K.A., Acharya C., McIntire W.E., Stevens R.C., Zhang Q., Harris A.L., Abagyan R., Yeager M. (2016). An electrostatic mechanism for Ca^2+^-mediated regulation of gap junction channels. Nat. Commun..

[37] Verselis V.K., Bennett M., Bargiello T.A. (1991). A voltage-dependent gap junction in *Drosophila melanogaster*. Biophys. J..

[38] Dahl G., Muller K.J. (2014). Innexin and pannexin channels and their signaling. FEBS Lett..

[39] Holcroft C.E., Jackson W.D., Lin W.-H., Bassiri K., Baines R.A., Phelan P. (2013). Innexins Ogre and Inx2 are required in glial cells for normal postembryonic development of the Drosophila central nervous system. J. Cell Sci..

[40] Xia L., Nie T., Lu F., Lu H., Shi X., Ren D., Lu J., Li X., Xu T., Cui B. (2024). Direct regulation of FNIP1 and FNIP2 by MEF2 sustains MTORC1 activation and tumor progression in pancreatic cancer. Autophagy.

[41] Huang X.-Y., Huang Z.-L., Zhang P.-B., Huang X.-Y., Huang J., Wang H.-C., Xu B., Zhou J., Tang Z.-Y. (2019). CircRNA-100338 Is Associated with mTOR Signaling Pathway and Poor Prognosis in Hepatocellular Carcinoma. Front. Oncol..

[42] Musgrove L., Russell F.D., Ventura T. (2024). Considerations for cultivated crustacean meat: Potential cell sources, potential differentiation and immortalization strategies, and lessons from crustacean and other animal models. Crit. Rev. Food Sci. Nutr..

[43] Wei X., Luo L., Chen J. (2019). Roles of mTOR Signaling in Tissue Regeneration. Cells.

[44] Xiao G., Li X., Yang H., Zhang R., Huang J., Tian Y., Nie M., Sun X. (2024). mTOR mutation disrupts larval zebrafish tail fin regeneration via regulating proliferation of blastema cells and mitochondrial functions. J. Orthop. Surg. Res..

[45] Liu J., Hu Y., Waller D.L., Wang J., Liu Q. (2012). Natural products as kinase inhibitors. Nat. Product. Rep..

[46] Thoreen C.C., Kang S.A., Chang J.W., Liu Q., Zhang J., Gao Y., Reichling L.J., Sim T., Sabatini D.M., Gray N.S. (2009). An ATP-competitive mammalian target of rapamycin inhibitor reveals rapamycin-resistant functions of mTORC1. J. Biol. Chem..

[47] Zhang X., Hu C., Chen T., Li P., Tan Y., Ren C., Wang Y., Jiang X., Ma B., Yin J. (2025). DNA methylation regulates growth traits by influencing metabolic pathways in Pacific white shrimp (*Litopenaeus vannamei*). BMC Genom..

[48] Greenberg M.V.C., Bourc’his D. (2019). The diverse roles of DNA methylation in mammalian development and disease. Nat. Rev. Mol. Cell Biol..

[49] Maamar M.B., Beck D., Nilsson E., McCarrey J.R., Skinner M.K. (2022). Developmental alterations in DNA methylation during gametogenesis from primordial germ cells to sperm. iScience.

[50] Andersen I.S., Reiner A.H., Aanes H., Aleström P., Collas P. (2012). Developmental features of DNA methylation during activation of the embryonic zebrafish genome. Genome Biol..

[51] Aguilar C., Gardiner D.M. (2017). DNA Methylation Dynamics Regulate the Formation of a Regenerative Wound Epithelium during Axolotl Limb Regeneration. PLoS ONE.

