# Supplementary material for: Innexin2 Regulates Limb Regeneration in Procambarus clarkii: Insights from Transcriptome and DNA Methylation Analyses
Source: Animals (Basel). 2026 Sep 24;16(19):3008. doi: 10.3390/ani16193008 (PMC13663385; doi:10.3390/ani16193008)
Supplement: Supplementary file 1 [file animals-16-03008-s001.zip › animals-4517380-supplementary.pdf]

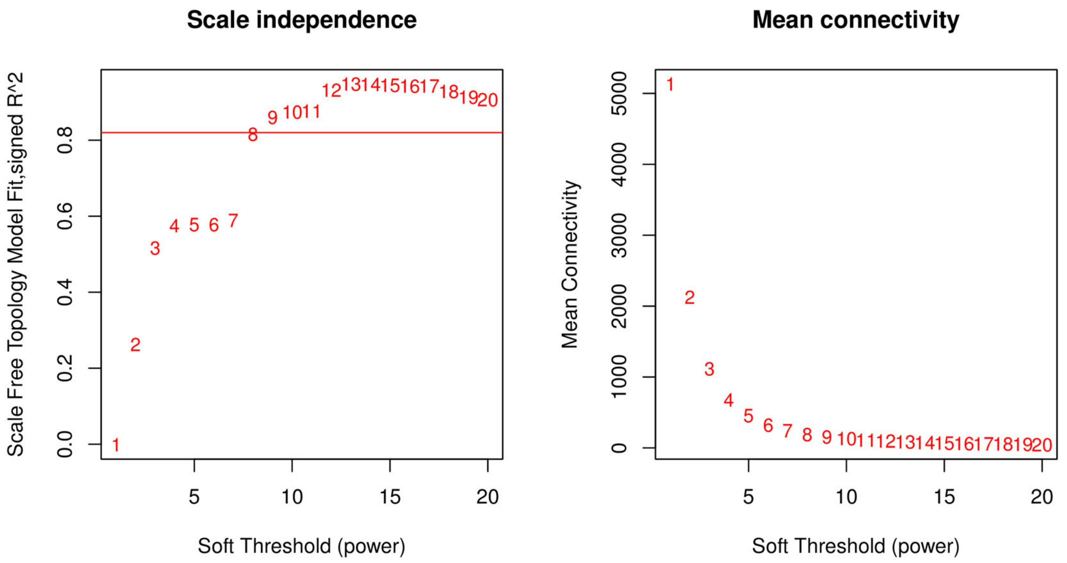

Figure S1. Correlation between the fitting index and soft threshold (left)

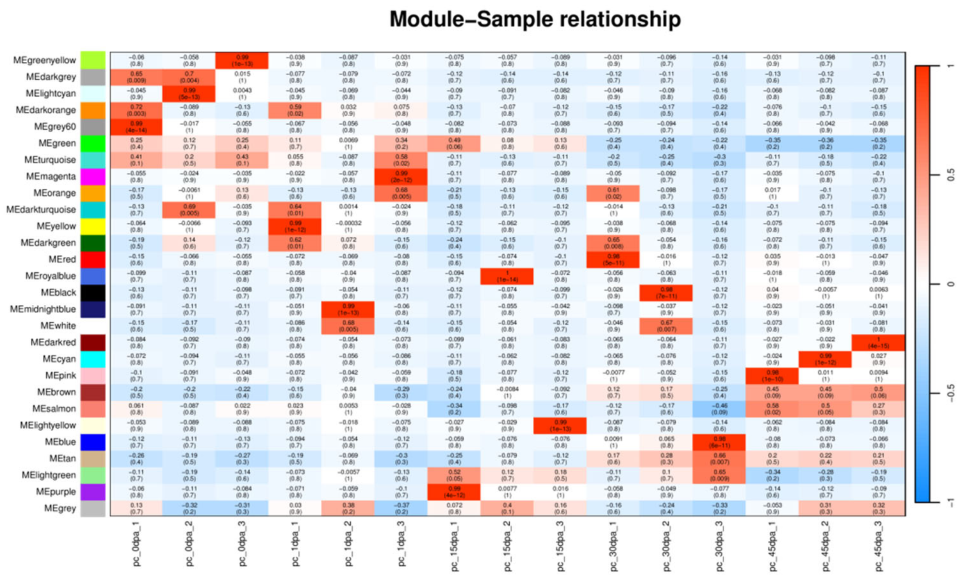

Figure S2. The relationship between common module characteristic genes and regeneration process was identified by WGCNA analysis. We determined the co-expression module of gene 227. With the time change of limb regeneration, the specific module of *Procambarus clarkii* showed strong correlation with different limb regeneration stages (0 dpa, 1 dpa, 15 dpa and 30 pad). Dpa: days post autotomy.

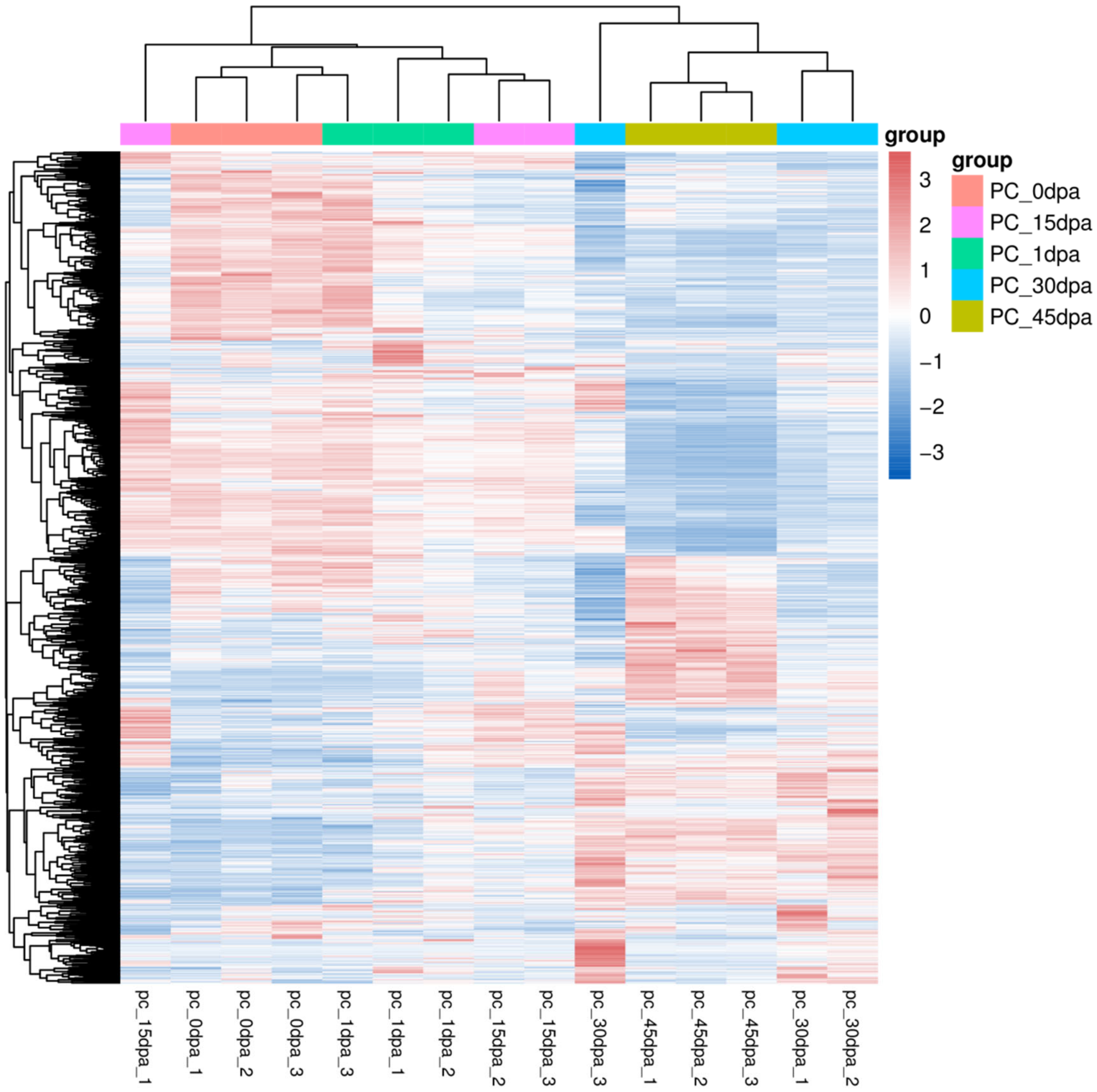

Figure S3. Differentially expressed gene *Procambarus clarkii* during limb regeneration. Thermogram of differentially expressed genes during limb regeneration.

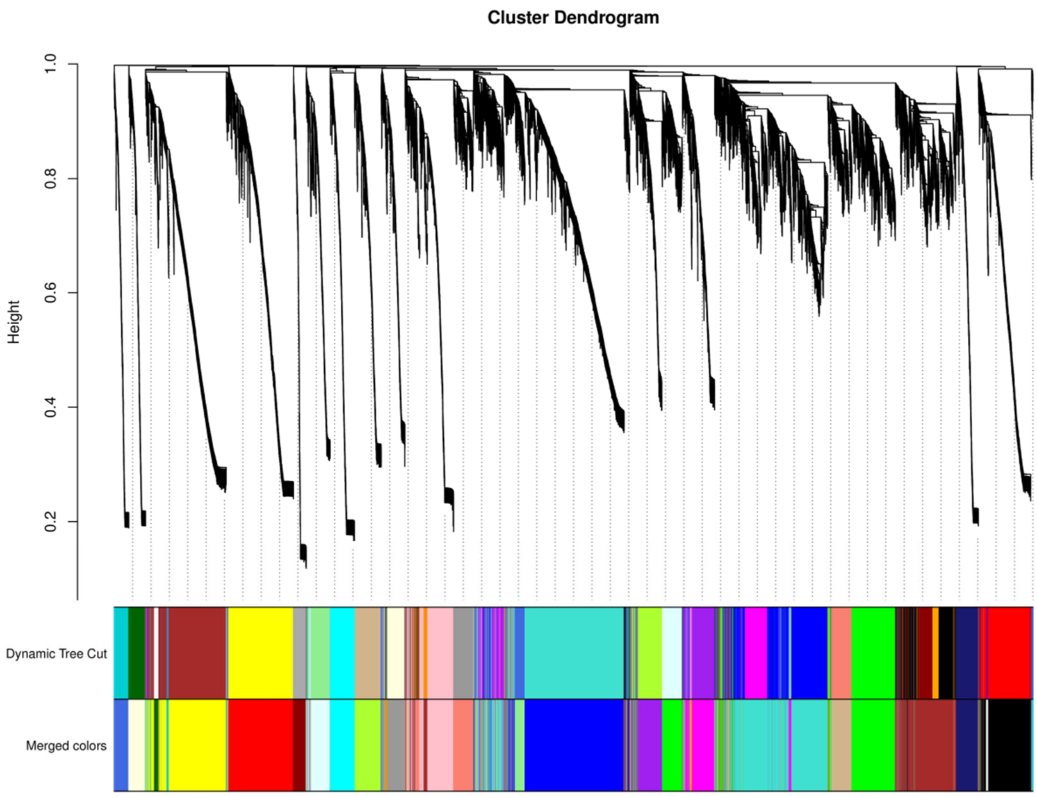

Figure S4. Identification of Cluster Tree Based on Co-expression Topology Overlapping Display Module by WGCNA . A total of 27 modules were identified, and different horizontal color bars represent different modules of co-expressed genes.

A

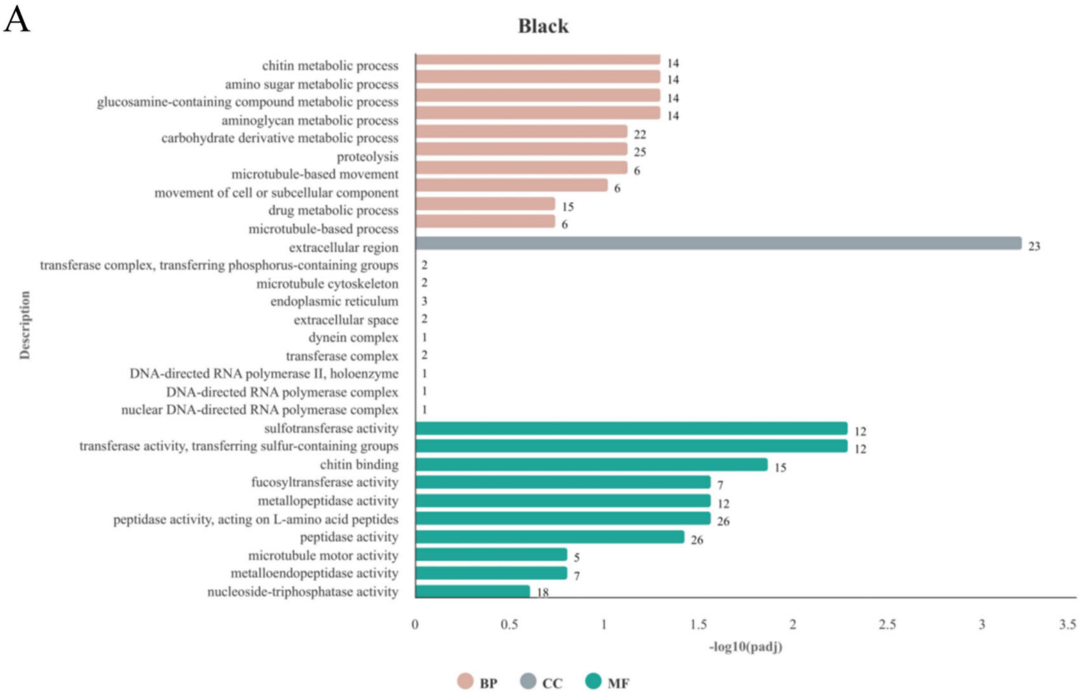

B

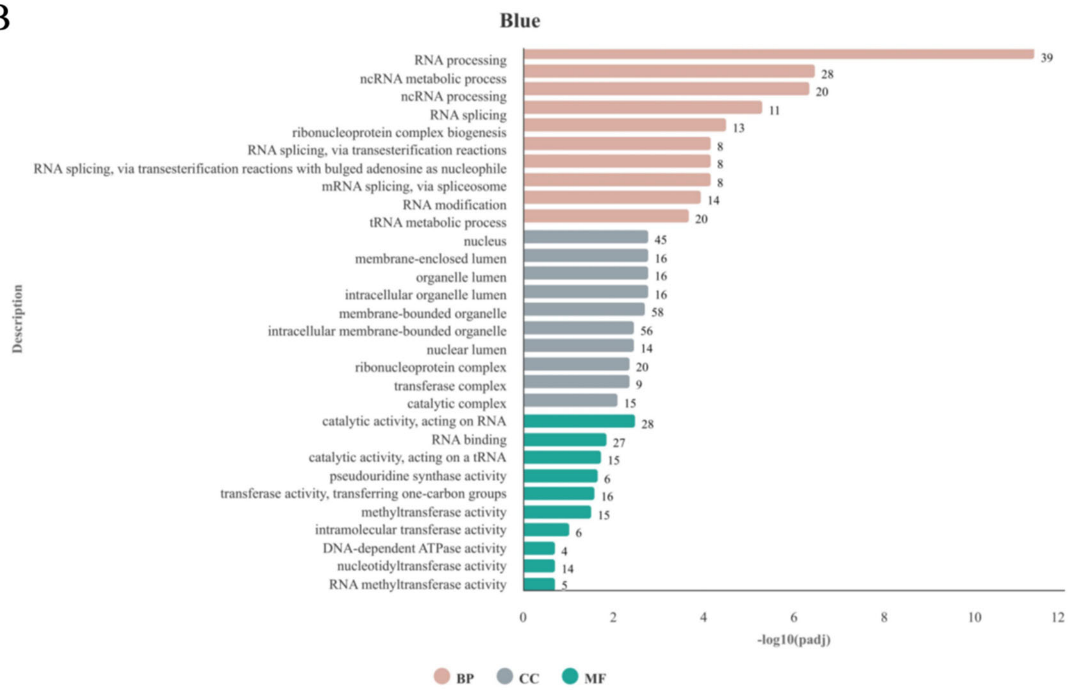

Figure S5. GO enrichment analysis for co-expressed genes in Module 1 (A) and Module 2 (B) from Figure 2 by WGCNA analysis.

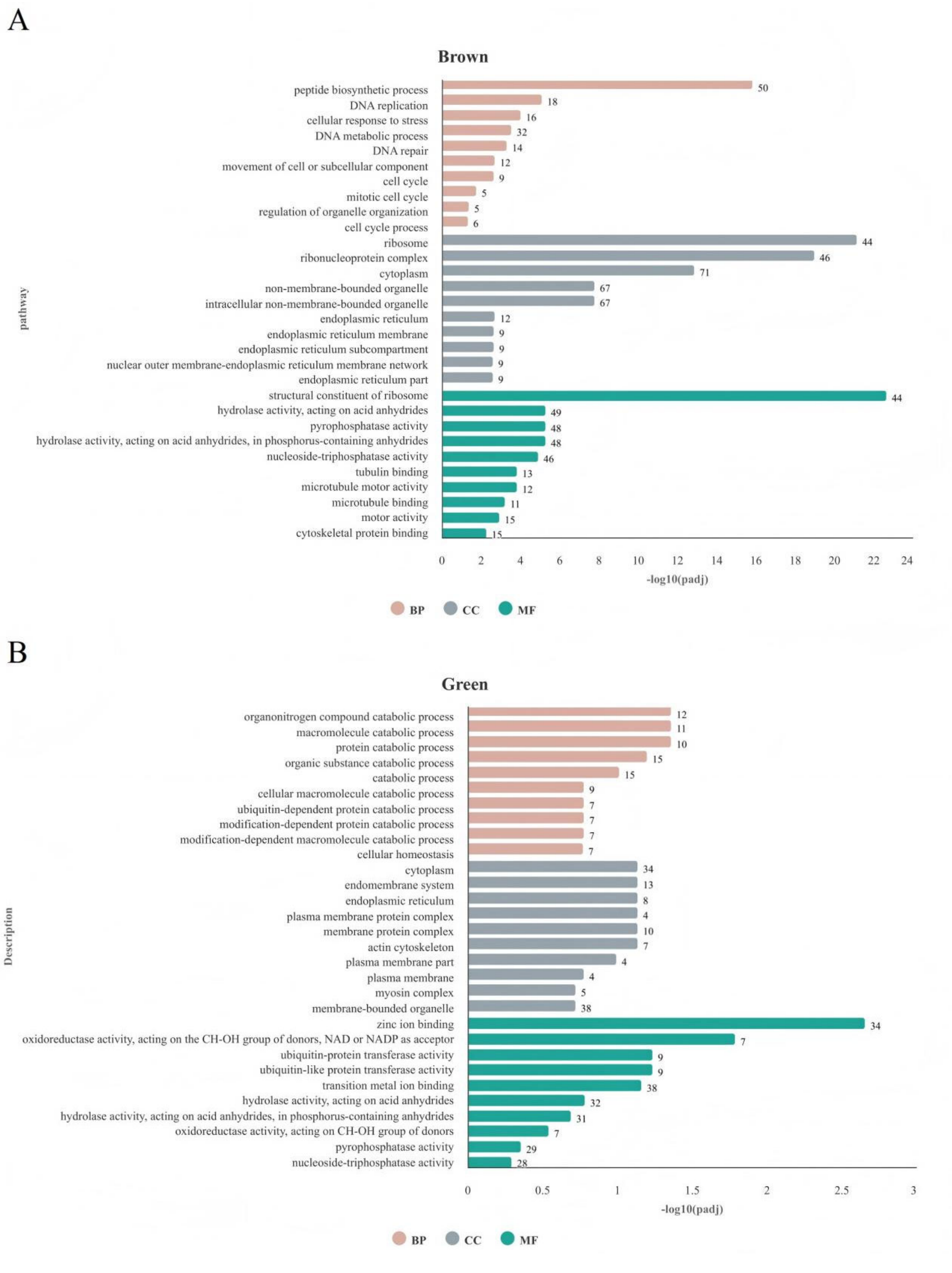

Figure S6. GO enrichment analysis for co-expressed genes in Module 3 (A) and Module 4 (B) from Figure 2 by WGCNA analysis.

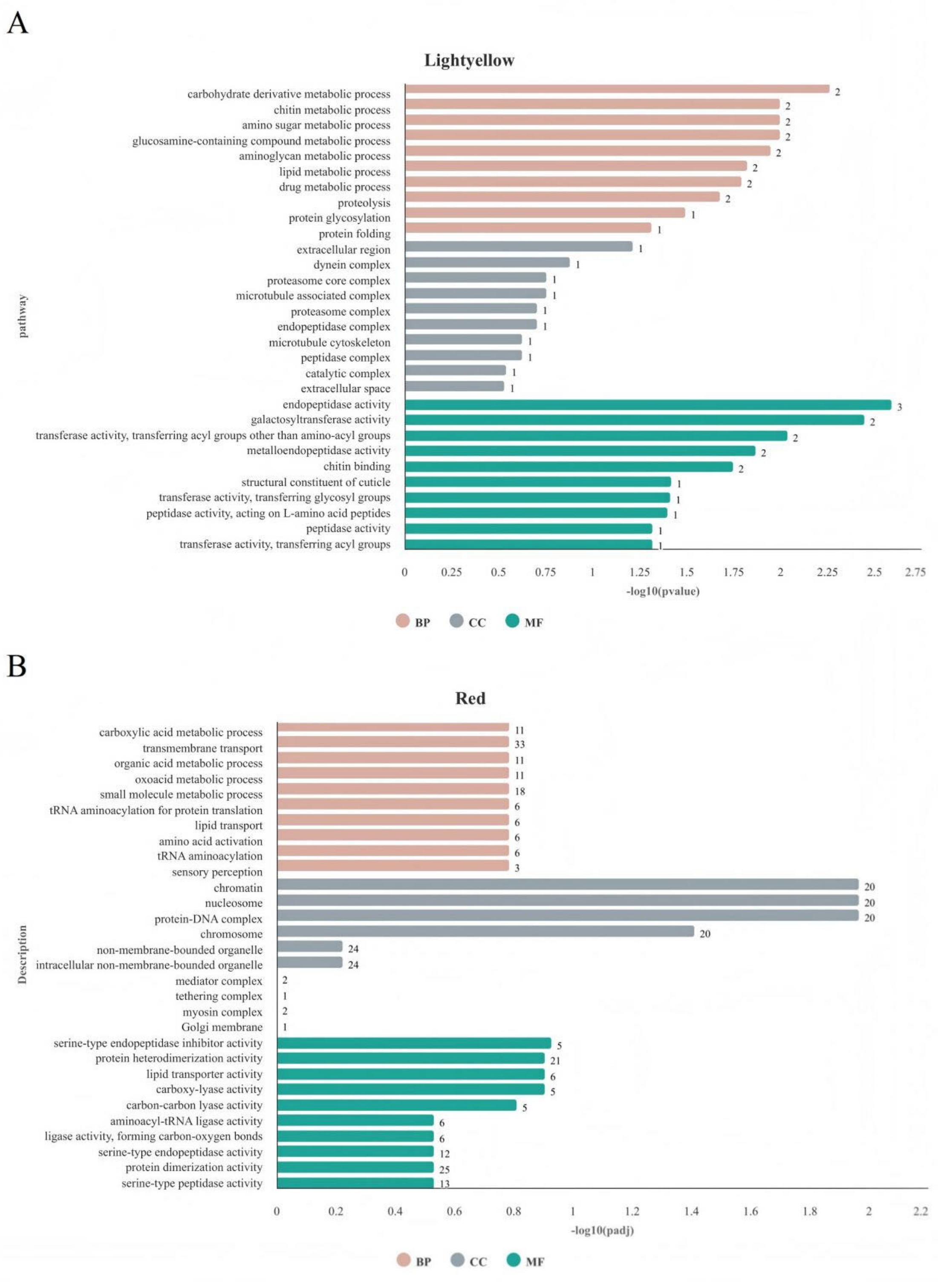

Figure S7. GO enrichment analysis for co-expressed genes in Module 5 (A) and Module 6 (B) from Figure 2 by WGCNA analysis.

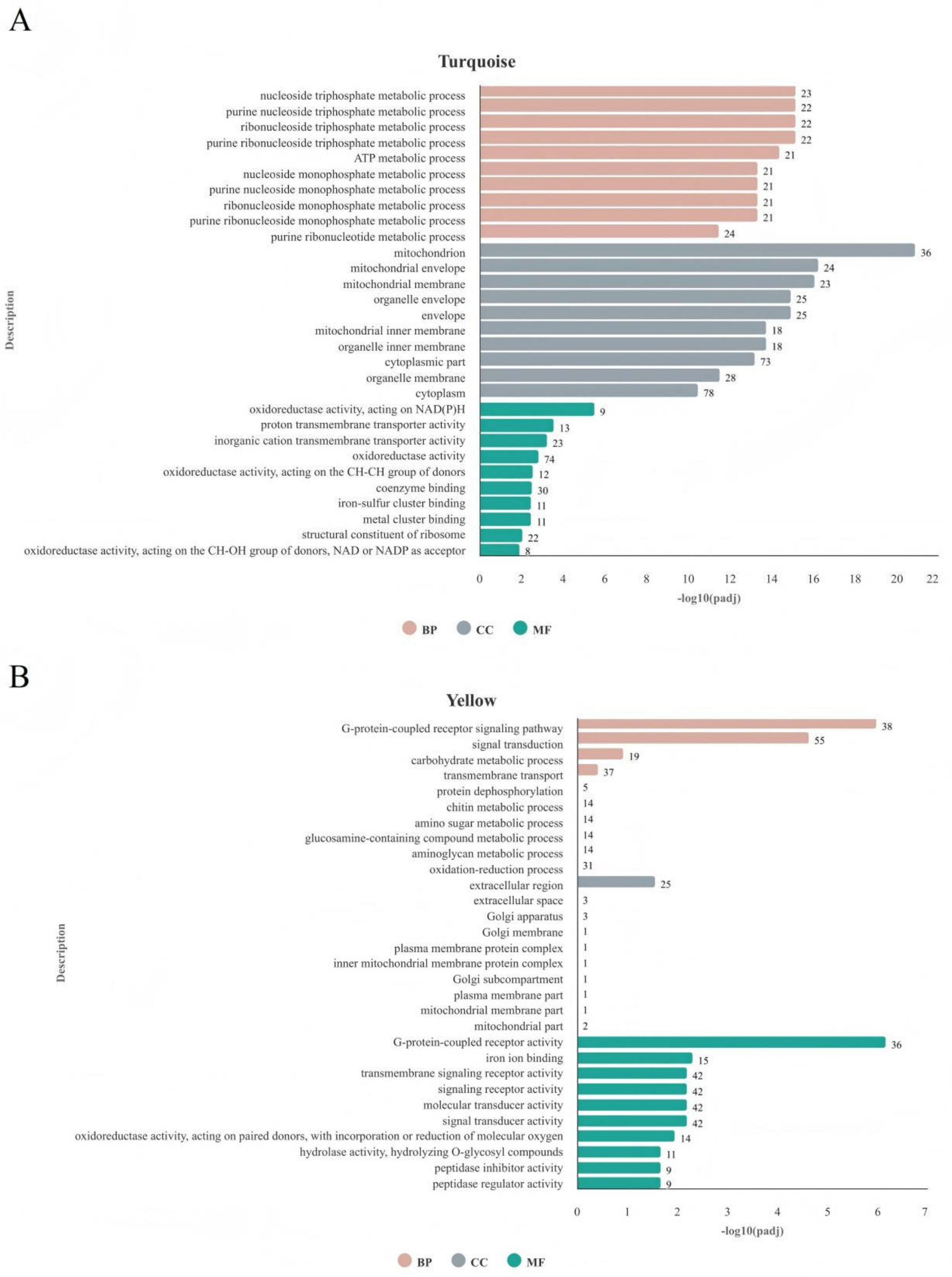

Figure S8. GO enrichment analysis for co-expressed genes in Module 7 (A) and Module 8 (B) from Figure 2 by WGCNA analysis.

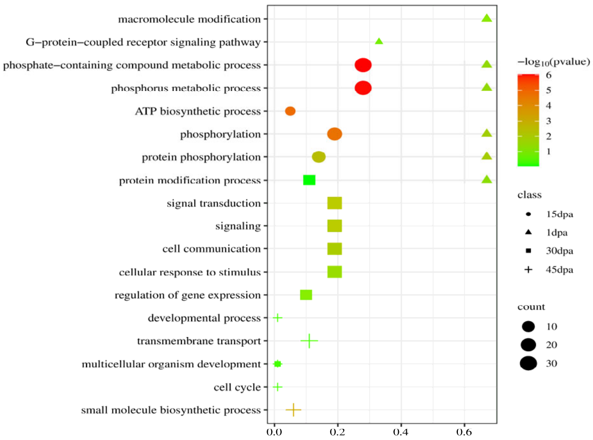

Figure S9. GO enrichment analysis is carried out on representative biological process categories of up-regulated genes. 1 dpa, 15 dpa, 30 dpa and 45 dpa during limb regeneration. dpa: day post autotomy.

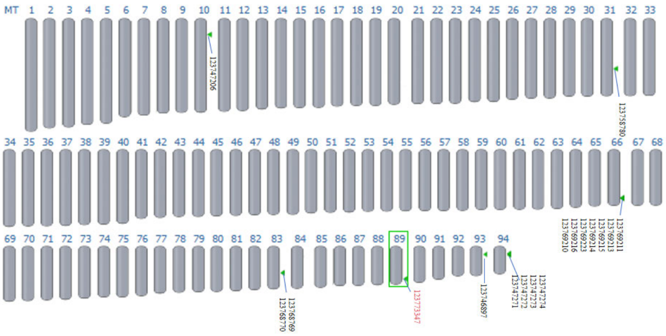

Figure S10. Chromosome distribution of 17 Innexin genes in the genome of *Procambarus clarkii*.

Table S1. Primer sequences used for RT-qPCR analysis for related genes in this study.

Primer Name	Primer Sequences (5'-3')
18S rRNA-F	GTCAGGTCATCACCATCGGCA
18S rRNA-R	CGGTCTCGTGAACACCAGCA
Inx2-F	CCCTCGCTACCTCTGGAA
Inx2-R	CTCGCACAGGAAGAACCTAA
